# Dimethyl Sulfoxide Promotes the Multiple Functions of the Tumor Suppressor HLJ1 through Activator Protein-1 Activation in NSCLC Cells

**DOI:** 10.1371/journal.pone.0033772

**Published:** 2012-04-17

**Authors:** Chi-Chung Wang, Sheng-Yi Lin, Yi-Hua Lai, Ya-Jung Liu, Yuan-Lin Hsu, Jeremy J. W. Chen

**Affiliations:** 1 Graduate Institute of Basic Medicine, Fu Jen Catholic University, Taipei, Taiwan; 2 Institutes of Biomedical Sciences and Molecular Biology, National Chung-Hsing University, Taichung, Taiwan; National Taiwan University Hospital, Taiwan

## Abstract

**Background:**

Dimethyl sulfoxide (DMSO) is an amphipathic molecule that displays a diversity of antitumor activities. Previous studies have demonstrated that DMSO can modulate AP-1 activity and lead to cell cycle arrest at the G1 phase. HLJ1 is a newly identified tumor and invasion suppressor that inhibits tumorigenesis and cancer metastasis. Its transcriptional activity is regulated by the transcription factor AP-1. However, the effects of DMSO on HLJ1 are still unknown. In the present study, we investigate the antitumor effects of DMSO through HLJ1 induction and demonstrate the mechanisms involved.

**Methods and Findings:**

Low-HLJ1-expressing highly invasive CL1–5 lung adenocarcinoma cells were treated with various concentrations of DMSO. We found that DMSO can significantly inhibit cancer cell invasion, migration, proliferation, and colony formation capabilities through upregulation of HLJ1 in a concentration-dependent manner, whereas ethanol has no effect. In addition, the *HLJ1* promoter and enhancer reporter assay revealed that DMSO transcriptionally upregulates *HLJ1* expression through an AP-1 site within the *HLJ1* enhancer. The AP-1 subfamily members JunD and JunB were significantly upregulated by DMSO in a concentration-dependent manner. Furthermore, pretreatment with DMSO led to a significant increase in the percentage of UV-induced apoptotic cells.

**Conclusions:**

Our results suggest that DMSO may be an important stimulator of the tumor suppressor protein HLJ1 through AP-1 activation in highly invasive lung adenocarcinoma cells. Targeted induction of HLJ1 represents a promising approach for cancer therapy, which also implied that DMSO may serve as a potential lead compound or coordinated ligand for the development of novel anticancer drugs.

## Introduction

Dimethyl sulfoxide (DMSO; (CH_3_)_2_SO) is an amphipathic molecule that has a highly polar domain and two apolar methyl groups, making it soluble in both aqueous and organic media [1]. Although its biological effects have not been clearly defined, it is used extensively in a variety of fields. It is commonly used as a very efficient solvent for water-insoluble compounds in biological studies and a cryoprotectant of cultured cells [2]. In addition, it is also popularly used as a vehicle for drug therapy for various diseases, including dermatological disorders [3], amyloidosis [4], gastrointestinal diseases [5], [6], traumatic brain edema [7], musculoskeletal disorders [8], pulmonary adenocarcinoma [9], rheumatologic diseases [10], and schizophrenia [11]. In particular, DMSO used in the treatment of interstitial cystitis has been approved by the United States Food and Drug Administration [12]. DMSO also had been used for treatment of leukemia for several years as it induces cellular differentiation, causing leukemia cells to lose their proliferative properties [13], [14]. Recent study also demonstrated that DMSO might induce cardiomyogenesis of P19CL6 embryonal carcinoma cells [15]. Furthermore, DMSO has been found to arrest the cell cycle of lymphoid cell lines at the G1 phase [16], [17], and it can effectively inhibit capillary tube formation through MMP-2 suppression [18].

With its high relapse and low cure rates, lung cancer is the most common cause of cancer mortality and incidence in the world [19]. Adenocarcinoma is the predominant histologic subtype of lung cancer in most countries, making up approximately 50% of all lung cancers [20]. In a previous study, we screened a series of human lung adenocarcinoma cell lines with varying invasion capabilities by microarray and identified a panel of metastasis-related genes including the human liver DnaJ-like protein (HLJ1, also known as DNAJB4) [21]. We subsequently demonstrated that HLJ1, a tumor suppressor in non-small cell lung cancer (NSCLC), can inhibit lung cancer proliferation, anchorage-independent growth, motility, invasion, tumorigenesis, and cell cycle progression. In addition, HLJ1 expression is correlated with reduced cancer recurrence and prolonged survival of NSCLC patients [22]. Furthermore, the endogenous transcriptional expression of *HLJ1* is upregulated via enhancer activator protein-1 (AP-1) binding to its promoter Yin-Yang-1 (YY1) with the coactivator p300 [23], [24]. Due to its tumor suppressor properties, HLJ1 is a potential target for anticancer therapy [25]. Importantly, HLJ1 was reported to promote UV-induced apoptosis through JNK and caspase-3 activation in NSCLC. Additionally, HLJ1 is a novel substrate of caspase-3 and is degraded at a late stage of apoptosis [26]. Therefore, clarifying the molecular mechanisms involved in HLJ1 upregulation may be important for anticancer therapy. Indeed, curcumin, an active component of the spice turmeric, has been reported to inhibit lung cancer cell invasion and metastasis through HLJ1 [27]. However, whether any other small molecules or chemicals can effectively modulate HLJ1 expression is still unknown.

Several studies have revealed that DMSO treatment can modulate AP-1 activity. For instance, it is involved in the suppression of ICAM-1 expression in a rat model of peritonitis sepsis [28] and in the respiratory syncytial virus (RSV)-induced production of IL-8 in A549 epithelial cells [29]. Because HLJ1 is transcriptionally upregulated by AP-1, we hypothesized that HLJ1 expression may be regulated by DMSO. In this report, we investigated the effect of DMSO on HLJ1 expression, the mechanism of DMSO-induced HLJ1 expression and the anticancer effects of DMSO in human lung adenocarcinoma CL1–5 cells. Moreover, we explored the effect of DMSO on apoptosis in CL1–5 cells exposed to UV stress.

## Methods

### Cell Culture

The human lung adenocarcinoma cell lines CL1–5 (with low HLJ1 expression, a gift from Dr. Cheng-Wen Wu) [30], NCI-H1299 (ATCC CRL-5803) and A549 (ATCC CCL-185) cells were maintained at 37°C in a humidified atmosphere of 5% CO_2_. Cells were cultured in RPMI-1640 medium (Life Technologies, Inc., Carlsbad, CA, USA) supplemented with 10% heat-inactivated fetal bovine serum (FBS; Life Technologies, Inc.) and 1% penicillin-streptomycin (Life Technologies, Inc.).

### Cell Cytotoxicity Assay

CL1–5 cells were seeded onto 96-well plates and cultured for 48 h with various concentrations of DMSO (0, 0.1%, 1%, 2%, 5%, and 10% v/v) (Sigma, St Louis, MO, USA). After culturing for various durations of time, cell viability was evaluated by the thiazolyl blue tetrazolium bromide (MTT) assay (Promega, Madison, WI, USA) using the multiwell scanning spectrophotometer Victor 3 (Perkin-Elmer, Boston, MA, USA) with the absorbance wavelength set at 570 nm.

### Immunofluorescent Staining

Cells cultured on 12 mm glass cover-slips were fixed for 15 min in PBS containing 4% paraformaldehyde and 2% sucrose and then permeabilized in PBS containing 0.3% Triton X-100 for 2 min. Cover-slips were incubated with primary antibody against F-actin at 4°C overnight followed by rhodamine-conjugated second antibody (Santa Cruz Biotechnology, Inc., Santa Cruz, CA. USA). 4′,6-diamidino-2-phenylindole (DAPI) was used as a counter stain for cell nuclei, and the cells were mounted onto slides and visualized by a confocal laser scanning microscope (Clsi; Nikon).

### Western Blot Analysis

Western blot analysis was used to examine the protein expression levels of JunD, JunB, Fra-1, and HLJ1 before and after DMSO treatment for 48 h. The detailed procedures were as described previously (Wang *et al*., 2005). The primary antibodies for JunD, JunB, Fra-1, and HLJ1 were purchased from Santa Cruz Biotechnology, Inc. β-actin or α-tubulin was used as the internal control for gel loading. The membranes were then washed three times with TBST followed by incubation with horseradish peroxidase (HRP)-conjugated secondary antibody (Santa Cruz Biotechnology, Inc) and detection using an enhanced chemiluminescence detection system (ECL, GE Healthcare, Piscataway, NJ, USA).

### Real-time Quantitative Reverse Transcription-PCR

The expression level of *HLJ1* before and after DMSO treatment was detected with real-time PCR on an ABI prism 7900 sequence detection system (Applied Biosystems, Carlsbad, CA, USA). The HLJ1 primers are as follows: forward primer QHLJ1-F, 5′-CCAGCAGACATTG- TTTTTATCATT-3′; reverse primer QHLJ1-R, 5′-CCATCCAGTGTTGGTACATTAATT-3′. TATA-box binding protein (TBP) was used as the internal control (GenBank X54993). The primers used for *TBP* mRNA are described previously [31]. The relative expression level of HLJ1 compared with that of TBP was defined as –ΔCT =  –[CT_HLJ1_–CT_TBP_]. The *HLJ1*/TBP mRNA ratio was calculated as 2^–ΔCT^ × K, in which K is a constant. Experiments were performed three times in triplicate.

### Transfection and Luciferase Reporter Assays

All transfections were performed in triplicate in 6-well plates, and the detailed protocol has been described in previous studies [23], [24]. Briefly, CL1–5 cells were seeded for 24 h prior to transfection. Plasmids were transfected into cells using Lipofectamine 2000 reagent according to the manufacturer’s instructions (Invitrogen, Carlsbad, CA, USA). The *HLJ1* promoter and enhancer luciferase reporter constructs [23], [24] and the control plasmid were cotransfected with a β-galactosidase construct, pSV-β-Gal (Promega, Madison, WI, USA). All constructs were confirmed by restriction endonuclease digestion and DNA sequencing. The ratio of the DNA amounts for luciferase reporter constructs versus β-galactosidase construct was 3∶1. The cells were incubated in transfection mixture for 4 h and then harvested after 44 h in culture with or without DMSO treatment. An aliquot of cell lysate (10–25 µl) was used to assay luciferase activity using a luciferase assay kit (Tropix, Inc., Bedford, MA, USA). Another aliquot (10–25 µl) was used to measure β-galactosidase activity using the Galacto-Light chemiluminescent assay kit (Tropix, Inc.). Luminescence was measured with a Victor 3 multilabel counter (Perkin-Elmer). Transfection efficiency was normalized with β-galactosidase activity. Each experiment was repeated at least three times in duplicate.

### Invasion and Migration Assays

The invasiveness of CL1–5 cells treated with various concentrations of DMSO was examined with transwell chambers (8-µm pore size; Corning Costar Corp., Cambridge, MA, USA) and transwell filters coated with Matrigel (BD Biosciences, Franklin Lakes, NJ, USA) as described previously [22]. The number of cells that attached to the lower surface of the polycarbonate filter was determined at 400× magnification under a light microscope. The migratory capability of the CL1–5 cells treated with or without DMSO was assessed using the wound healing approach as described previously [22]. The number of cells migrating into the cell-free zone was determined by counting under a light microscope. All experiments were performed in triplicate.

### siRNA Transient Transfection

Desalted siRNA duplexes were synthesized by Qiagen (Valencia, CA, USA) and were annealed following a standard protocol. The scramble and *JunD* siRNAs used in this assay were described previously [23], [27]. siRNAs were transfected using the RNAiFect Transfection Reagent (Qiagen) according to the manufacturer’s instruction. After 24 h, cells were treated with various concentrations of DMSO for another 24 h.

### Cell Proliferation and Colony Formation Assay

CL1–5 cells were seeded onto 96-well plates (3 × 10^3^ cells/well). RPMI-1640 complete medium with various concentrations of DMSO (0–2%) were replaced the next day, beginning the day after seeding. After culturing for various durations (24, 48, 72, and 96 h), cell proliferation was evaluated by thiazolyl blue tetrazolium bromide (MTT) assay according to the manufacturer’s protocol (Chemicon). All experiments were performed in two independent experiments (n  =  6 per group). To determine anchorage-dependent colony formation, CL1–5 cells were seeded at 200 cells per well in RPMI-1640 complete medium in 6-well plates. The plates were incubated for 7–10 days and then stained with 0.1% crystal violet. To determine anchorage-independent growth, 6-well plates were precoated with 0.7% agarose in RPMI-1640 with 10% FBS, and CL1–5 cells were seeded at 1.5 × 10^3^ cells per well in 0.35% agarose/RPMI-1640 with 10% FBS. The plates were incubated for 1 month and then stained with 0.1% crystal violet. Colonies with a diameter greater than 1 mm were counted under an inverted microscope. The colony formation assays were assessed in two independent experiments in triplicate.

### UV Irradiation and Flow Cytometry

A flow cytometer was employed to determine the percentage of the sub-G1 cell population, which indicates UV-induced apoptosis. UV-irradiation was followed mainly as described previously [26]. Briefly, CL1–5 cells were seeded at a density of 8×10^5^ cells/10 cm dish in medium with 10% FBS. After overnight preincubation at 37°C, cells were pretreated with 1% or 2% of DMSO for 24 h and then exposed to 10 J/m^2^ of UV irradiation (254 nm) (BIO-LINK BLX-254, Vilber Lourmat, France). After recovery for 48 h, each sample was washed with ice-cold PBS, harvested and fixed in 70% (v/v) ethanol for 2 h at –20°C. Cells were treated with RNaseA and stained with 25 µg/ml propidium iodine. Samples were analyzed using a Cytomics^TM^ FC500 flow cytometer (Beckman Coulter, Brea, CA, USA), according to the manufacturer’s protocol. Cytometric data were analyzed with WinMDI 2.8 software. A minimum of 10,000 events were examined. Experiments were performed three times in duplicate.

### Statistical Analysis

All experiments were performed three times in triplicate and significant differences were analyzed by ANOVA (Excel, Microsoft). Data were considered statistically significant at *P* < 0.05. Where appropriate, results are presented as the means ± SD.

## Results

### Cytotoxic Effects of DMSO on CL1–5 Cells

**Figure 1:**
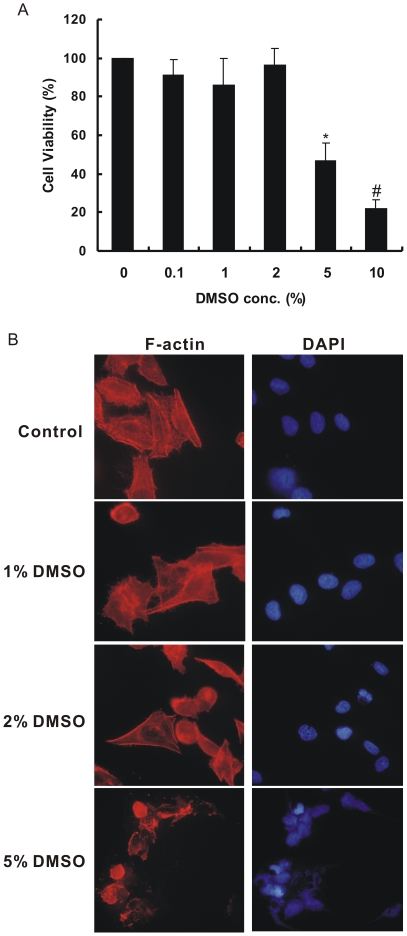
Effects of various concentrations of DMSO on CL1–5 cell viability. (A) The viability of lung adenocarcinoma CL1–5 cells cultured with various concentrations of DMSO by MTT assay. *P < 0.0005, #*P* < 0.0001 significantly different from the vehicle-treated control. (B) CL1–5 cells treated with or without DMSO were stained with phalloidin-tetramethylrhodamine isothiocyanate conjugate to detect F-actin (red). DMSO-treated cells exhibit fewer filopodia fibers, whereas the high concentration of DMSO (5%) leads to the cell death. The cell nuclei were counter stained with DAPI. The image magnification is×400.

The effects of dimethyl sulfoxide on the viability of CL1–5 cells are shown in Figure 1. Data presented in Figure 1A indicate a dose response relationship with respect to the cytotoxicity of DMSO. On *in vitro* cultured CL1–5 cells, low and moderate DMSO treatment did not cause cytotoxicity up to a 2% (v/v) concentration. However, at the high concentrations of DMSO (≥ 5%), approximately 50% or more reduction in cell viability was observed. The cells became round, shrunk and lost their stress fibers and normal morphology after 5% DMSO treatment, as shown in Figure 1B. Thus, we chose the doses no more than 2% DMSO for further functional assays.

### DMSO Stimulates HLJ1 Expression in Various Lung Adenocarcinoma Cells

**Figure 2:**
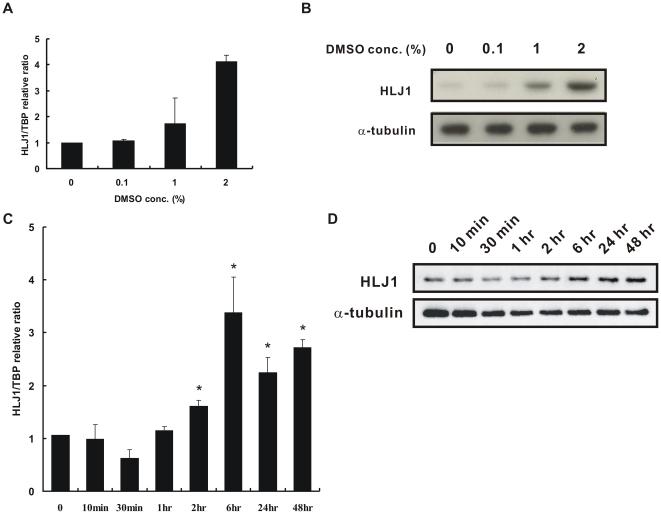
DMSO induces HLJ1 expression in concentration-dependent and time-dependent manners. (A) Real-time quantitative RT-PCR reveals that HLJ1 mRNA expression was induced by DMSO in a concentration-dependent manner (0.1–2%, v/v) after 48 h incubation. (B) Concentration-dependent DMSO-induced HLJ1 expression at the protein level was confirmed by Western blot analysis. α-tubulin was a control for protein loading and transfer. (C) Time-dependent DMSO-induced HLJ1 expression at the mRNA level was verified by real-time quantitative RT-PCR. The results indicated that the mRNA level of HLJ1 was significantly increased after 2% DMSO treatment for 2 h. *P < 0.05, significantly different from the vehicle-treated control. (D) The protein expression level of HLJ1 after 2% DMSO treatment at the indicated time was detected by Western blot analysis. The result indicated that HLJ1 protein expression was time-dependent and was sustained with DMSO treatment for 48 h. α-tubulin was a control for protein loading and transfer.

To determine whether DMSO induces expression of the tumor suppressor HLJ1, different lung adenocarcinoma cells were treated with various doses of DMSO, and then real-time QRT-PCR and/or Western blot analysis were performed (Figure 2 and Figure S1). As shown in Figures 2A and 2B, DMSO increased the mRNA and protein expressions of HLJ1 in CL1–5 cells in a concentration-dependent manner. HLJ1 expression was induced at DMSO dosages between 1% and 2%. The HLJ1 expression level was increased approximately 2- and 4-fold with 1% and 2% DMSO, respectively. In addition, DMSO also induced HLJ1 protein expression levels in A549 and H1299 cells below 1% DMSO dosage in a concentration-dependent manner (Figure S1). Taken together, these findings indicate that the HLJ1 induction by DMSO may be a common phenomenon among different lung adenocarcinoma cell lines. To rule out the possibility that the induction of HLJ1 was due to solvent stress effect, CL1–5 cells were treated with various doses of ethanol and the HLJ1 expression levels were determined using Western blot analysis. As shown in Figure S2, solvent ethanol has no effect on the induction of HLJ1. The non-cytotoxic 2% DMSO was chosen for further time course experiments. The results showed that DMSO induced mRNA and protein expression levels in a time-dependent manner (Figures 2C and 2D). The HLJ1 mRNA and protein levels increased after 2 h of DMSO treatment and reached a maximum level at 6 h (*P* < 0.05), which was sustained for up to 48 h at the protein level.

### The AP-1 Site within the HLJ1 Enhancer is Responsible for HLJ1 Induction by DMSO

**Figure 3:**
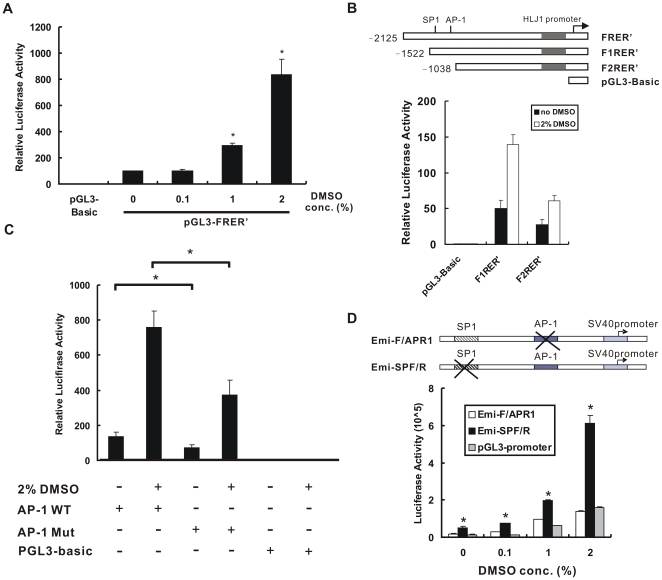
Up-regulation of *HLJ1* transcriptional activity by DMSO. (A) Relative luciferase activities of pGL3-FRER’ (containing the full-length HLJ1 promoter and enhancer) were determined in CL1–5 cells treated with various concentrations of DMSO. The results were correlated with the luciferase activity from cotransfected pSV-β-Gal cells and expressed as relative luciferase activity. *P < 0.05, significantly different from the vehicle-treated control. (B) 5′ deletion constructs of the HLJ1 promoter were represented in the upper panel. Relative luciferase activities of pGL3-F1RER’ and pGL3-F2RER’ (containing the full-length HLJ1 promoter with or without the AP-1 site, respectively) were determined in CL1–5 cells treated with or without 2% DMSO. (C) The mutation analysis of the AP-1 site within the *HLJ1* minimum enhancer indicated that the AP-1 site is critical for DMSO-induced *HLJ1* promoter activity. *P < 0.05, wild-type AP-1 site constructs compared with mutants. These results are representative of three independent experiments. (D) Further AP-1 site mutation analysis of the *HLJ1* minimum enhancer was confirmed by luciferase assay. The mutant derivative of the enhancer region where the motif was substituted was fused to the pGL3-promoter vector. Emi-F/APR1, the constructs of site-directed mutagenesis of the AP-1 site; Emi-SPF/R, the constructs of site-directed mutagenesis of the SP1 site. The results were correlated with the luciferase activity from cotransfected pSV-β-Gal cells and expressed as relative luciferase activity. *P < 0.05, compared with pGL3-promoter control. DMSO increases *HLJ1* enhancer activity through the AP-1 site in a concentration-dependent manner.

We have previously determined that the YY1 and AP-1 binding sites in the *HLJ1* promoter and enhancer regions play a major role in HLJ1 transcriptional regulation [24]. To study whether DMSO could regulate *HLJ1* expression through the promoter and/or enhancer regions of *HLJ1*, several constructs with deletions or mutations were used [24]. The full-length of the *HLJ1* promoter with enhancer (pGL3-FRER’) was used to show that DMSO could induce luciferase activity in a concentration-dependent manner (Figure 3A). In addition, the luciferase activity of the HLJ1 promoter was increased more effectively with an intact AP-1 site (pGL3-F1RER’) than with an AP-1 site deletion (pGL3-F2RER’) after 2% DMSO treatment (Figure 3B). Moreover, DMSO had less effect on the full-length construct with the mutated AP-1 site within the *HLJ1* enhancer. When compared with the wild-type construct (FRER’; AP-1 WT), the mutant of the AP-1 binding site (AP-1 Mut) significantly reduced *HLJ1* full-length promoter activity to 49% of WT activity after 2% DMSO treatment (Figure 3C). These results indicate that the AP-1 site is critical for DMSO-induced *HLJ1* promoter activity. Furthermore, site-directed mutagenesis analysis indicated that DMSO could significantly activate *HLJ1* enhancer activity through the AP-1 site and not the SP1 site in CL1–5 cells in a concentration-dependent manner (*P* < 0.05; Figure 3D).

**Figure 4:**
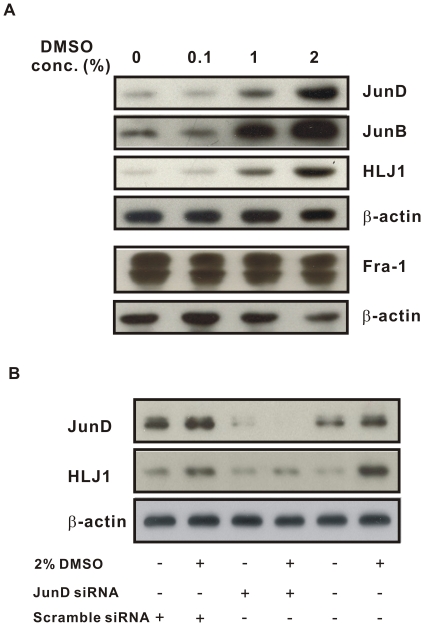
DMSO induces HLJ1 expression through AP-1. (A) Western blotting analysis shows that the expressions of the components of AP-1 (JunD and JunB) are regulated by DMSO in a dose-dependent manner, whereas the expression of Fra-1 shows no change with DMSO treatment. (B) CL1–5 cells transfected with JunD siRNA or the scrambled siRNA control were treated with or without 2% DMSO for 24 h and then analyzed by Western blotting. Knock-down of JunD decreased DMSO-induced HLJ1 expression. Data are representative of at least two independent experiments with β-actin used as the internal control.

There are several protein components that make up the AP-1 complex including c-Jun, c-Fos, JunD, JunB, Fra-1, Fra-2, and FosB. We have previously shown that JunD and JunB can upregulate HLJ1 expression [24]. While studying the effects of DMSO on the AP-1 subfamily, we found that JunD and JunB were also significantly induced by DMSO in CL1–5 cells (Figure 4A). However, the expression levels of Fra-1 are not affected significantly by DMSO over the concentration range used (0.1–2%). To determine the role of JunD in DMSO-induced HLJ1 expression, JunD siRNA was transfected into DMSO-treated CL1–5 cells to knock down JunD expression as described previously [27]. Figure 4B shows that pretreatment with JunD siRNA was able to reduce DMSO-induced HLJ1 expression, whereas the scramble siRNA had no effect. However, the expression level of the transcription factor YY1 was not changed with or without DMSO treatment (data not shown).

### DMSO Inhibits Cancer Cell Migration and Invasion Through Induction of HLJ1

**Figure 5:**
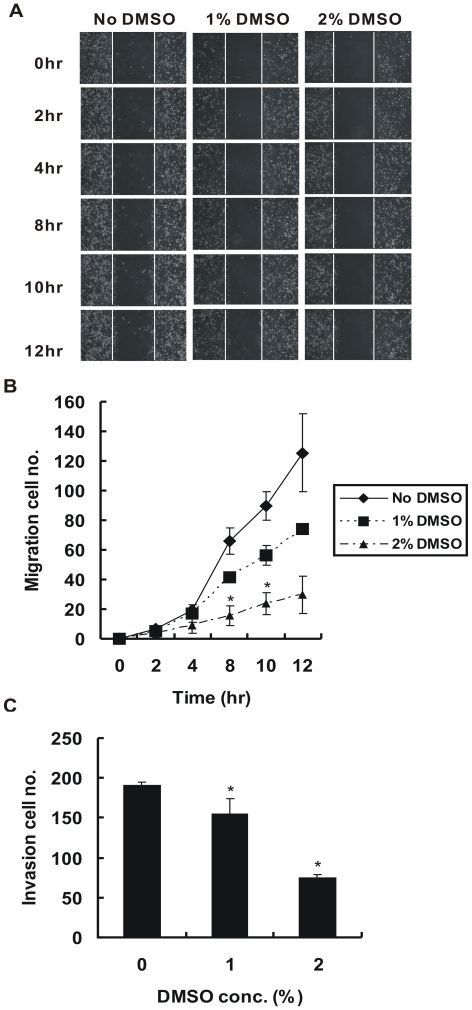
Suppression of *in vitro* migration and invasion capabilities of CL1–5 cells by DMSO treatment. (A) DMSO decreases cancer cell migration ability, as assessed by a scratch wound healing assay. The track was photographed immediately and after various times as indicated after wounding. The number of cells that migrated into the cell-free zone was evaluated (B). The results are expressed as the total number of the migrated cells. These results are representative of two independent experiments performed in triplicate. *P < 0.05, significantly different from the vehicle-treated control at the indicated time. (C) Invasiveness of CL1–5 cells treated with or without DMSO was evaluated by the Matrigel-based transwell invasion assay. Columns, mean derived from three separate experiments done in triplicate; bars, SD. *P < 0.05, significantly different from the vehicle-treated control.

CL1–5 is a highly invasive cancer cell line both *in vitro* and *in vivo* [29]. In our previous study, we demonstrated that HLJ1 expression levels were markedly lower in CL1–5 cells than in less invasive cells. Additionally, overexpression of HLJ1 in CL1–5 cells can inhibit cancer cell invasion and migration [22]. As we determined that 2% DMSO can induce HLJ1 expression in CL1–5 cells, we next investigated the effects of 2% DMSO on migration and invasion by CL1–5 cells in the wound-healing and Matrigel-based transwell assays. The results revealed that DMSO (1–2%) significantly inhibits CL1–5 cancer migration in a concentration- and time-dependent manner (Figures 5A and 5B). We found that the migration capability of CL1–5 cells was decreased significantly after HLJ1 induction (6 hr treatment of 2% DMSO) (*P* < 0.05). Furthermore, the Matrigel-based transwell assay indicated that DMSO could also significantly inhibit CL1–5 cell invasion, especially with 2% DMSO, which induces higher HLJ1 expression (*P* < 0.05; Figure 5C). In our previous study, we showed that the reduction in cancer cell invasion is mediated, at least in part, through upregulation of E-cadherin expression [23]. Thus, we used real-time RT-PCR to examine whether DMSO treatment could also affect the expression of E-cadherin. The results showed that the expression of E-cadherin was upregulated by DMSO; however, Slug expression was down-regulated (unpublished data).

**Figure 6:**
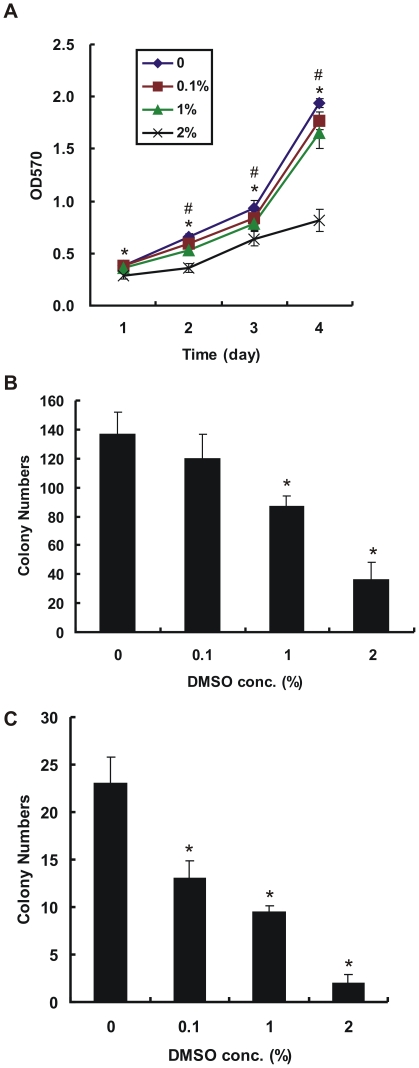
Inhibition of proliferation and colony formation of CL1–5 cells by DMSO treatment. (A) The proliferation of CL1–5 cells treated with various concentrations of DMSO was assessed by MTT assay (n  =  6 per group). #*P* < 0.05, 0.1–1% DMSO treatment significantly different from the no-treated control. *P < 0.05, 2% DMSO treatment significantly different from the no-treated control. (B) DMSO inhibits the anchorage-dependent colony formation of CL1–5 cells (n  =  4 per group). *P < 0.05, significantly different from the no-treated control. (C) Anchorage-independent colony formation of CL1–5 cells was determined by soft agar assay (n  =  4 per group). DMSO inhibits the anchorage-independent colony formation of CL1–5 cells. *P < 0.05, significantly different from the no-treated control.

### DMSO Suppresses Cancer Cell Proliferation and Colony Formation

We next examined whether CL1–5 cancer cell proliferation activity was affected by DMSO treatment. The results showed that DMSO can inhibit cancer cell proliferation in a dose-dependent manner, as shown by MTT assay (Figure 6A). The inhibitory effects of DMSO on anchorage-dependent and -independent growth are shown by significantly reduced colony formation of CL1–5 cells treated with 1% and 2% DMSO compared with no treatment (*P* < 0.05; Figures 6B and 6C). Previous data demonstrated that HLJ1 expression can inhibit lung cancer cell proliferation and anchorage-independent growth [22], and our study shows that DMSO can stimulate HLJ1 expression in CL1–5 cells. Thus, we conclude that the inhibitory effects of DMSO on cell proliferation and colony formation are mediated by induction of HLJ1.

**Figure 7:**
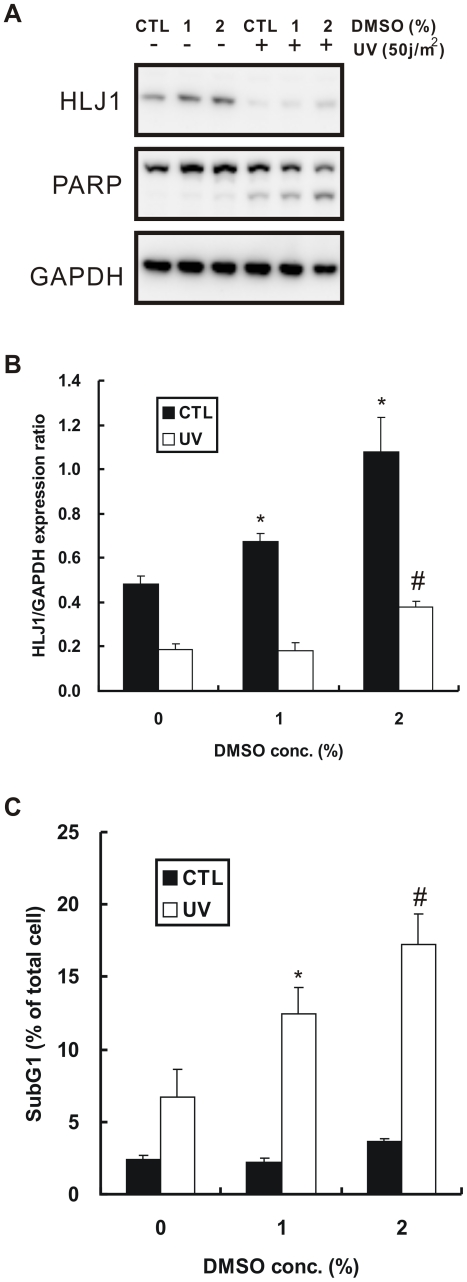
Effect of DMSO treatment on UV-induced apoptosis. (A) DMSO enhances PARP cleavage caused by UV-irradiation. After DMSO and UV treatments, CL1–5 cells were analyzed by Western blotting with anti-PARP and HLJ1 antibodies. GAPDH was used as an internal control. (B) Quantitative results of three independent western blotting analyses are shown. *P < 0.05, #*P*  =  0.014, compared with DMSO untreated control. (C) DMSO increases UV-induced apoptosis. CL1–5 cells were pretreated with DMSO and exposed to UV-irradiation. After a 48 h recovery period, the percentages of apoptotic cells were quantified by flow cytometry and presented as the means ± S.D. of three experiments. *P < 0.05, #*P* < 0.01, significantly different from the cells pretreated without DMSO and exposed to UV irradiation.

### DMSO Increases UV-induced Apoptosis Through Enhancing the Expression of HLJ1

Our previous study showed that induced expression of HLJ1 in CL1–5 cells can promote UV-induced apoptosis through activation of JNK and caspase-3 [26]. To determine the effects of DMSO on UV-induced apoptosis, CL1–5 cells were pretreated with 1% or 2% DMSO and then exposed to UV light. Western blot analyses showed that UV exposure led to a dramatic decrease in the HLJ1 expression level, even after DMSO pretreatment, compared with the non-treated control (Figure 7A). However, the decrease in HLJ1 was accompanied by increased cleavage of PARP, an important substrate of caspase-3, in a dose-dependent manner. In the control group, DMSO treatment increased HLJ1 expression in a dose-dependent manner (Figure 7B; *P* < 0.05). After UV exposure, HLJ1 expression increased only with 2% DMSO pretreatment (Figure 7B; *P*  =  0.014). As shown in Figure 7C, CL1–5 cells treated with DMSO alone had a slightly positive change in percent of apoptotic cells, but pretreatment with DMSO led to a significant increase in the percentage of UV-induced apoptotic cells compared to that of cells not pretreated with DMSO (*P* < 0.05 ).

## Discussion

Dimethyl sulfoxide is a by-product of the wood industry that has been used extensively as a common solvent and pharmaceutical agent. It is predominantly used as a topical analgesic and as a vehicle for drug therapy because of its antioxidant and anti-inflammatory effects [5]–[7], [32]. Many reports have recommended its use in pain, inflammation, interstitial cystitis, and cancer treatments [13], [33], [34]. However, the FDA has only approved its use for the symptomatic relief of patients with interstitial cystitis [12]. Some reports have demonstrated the putative mechanisms or target molecules regulated by DMSO that mediate its anti-inflammation properties and the ability to scavenge reactive oxygen species. In addition, it was reported that DMSO could induce widespread apoptosis at concentrations of 0.5% and 1% in a rat hippocampal culture [35]. However, in our study, cell viability was markedly decreased with 5% DMSO treatment. Thus, we chose the doses no more than 2% DMSO for further functional assays.

Recent data reported that DMSO upregulates tumor suppressor PTEN expression through NF-κB activation in human promyelocytic leukemic HL60 cells [36]. In this study, our data show that the expression of another tumor suppressor protein HLJ1 is also upregulated by DMSO, both at the mRNA and protein levels in dose- and time-dependent manners. Our previous studies showed that HLJ1 is regulated by AP-1 through the enhancer region of *HLJ1* [23], [24]. AP-1 is a nuclear transcription complex which is composed of Jun-Jun and Jun-Fos protein dimers that are involved in various biological mechanisms including normal cell growth and tumor development [37]. Chang et al. reported that DMSO, which serves as an antioxidant, can inhibit sepsis-induced activation of NF-κB and AP-1, resulting in the suppression of ICAM-1 gene expression [28]. Indeed, our results confirm that the AP-1 family members JunD and JunB are upregulated by DMSO in a dose-dependent manner in lung adenocarcinoma CL1–5 cells. However, another AP-1 stimulator, phorbol ester, could not induce the expression levels of JunD and JunB in CL1–5 cells after 24 hr treatment and fail to leading to HLJ1 induction (data not shown). JunD siRNA significantly but partially blocked this DMSO-induced HLJ1 expression. As JunB is also enhanced by DMSO, knockdown of only JunD is not sufficient to block overall HLJ1 induction. These results are consistent with our previous study [24]. There are three potential AP-1 binding sites within the HLJ1 full-length promoter, we could not rule out the possibilities that other AP-1 sites may have some effects on the HLJ1 promoter activity. In addition, other unknown transcription factors may also play some roles in this transcriptional regulation. Further studies will be needed to clarify all of these possibilities mentioned above.

Several mechanisms of the anti-tumor activity of DMSO have been reported. For example, DMSO has anti-angiogenic effects via inhibition of MMP-2 production [18], could mimic thalidomide to suppress choriocapillary endothelial cell proliferation [38], and could inhibit vascular smooth muscle cell (VSMC) proliferation and migration [39]. However, this is the first study to show that DMSO can inhibit lung adenocarcinoma cell migration, invasion, proliferation, and colony formation through induction of tumor suppressor HLJ. In recent years, ruthenium-based molecules have emerged as potential antitumor and antimetastatic agents. For instance, NAMI-A, [ImH][trans-RuCl4(DMSO)(Im)] (Im = imidazole), a Ru(III) complex with DMSO and imidazole coordinated to the ruthenium, has finished the phase I study [40]. In acidic environment, the hydrolysis of the ligand DMSO in NAMI-A may affect its antimetastatic activity [41], [42]. Whether the antimetastatic effect produced by NAMI-A through DMSO hydrolysis is mediated by HLJ1 induction needs further investigation. Furthermore, consistent with our data, a previous study demonstrated that DMSO-treated murine alveolar carcinoma line 1 cells form significantly fewer metastases than parental cell lines [43]. In contrast, Takenaga reported that treatment of Lewis lung carcinoma cells (P-29) with DMSO enhanced their metastatic potential [44]. This result is very different from that of others and ours; one possible reason is that the reports studied different cell lines. The different cell contents and genetic backgrounds may affect the results of these studies.

A previous study observed that DMSO could induce apoptosis in murine lymphoma cells through mitochondrial dysfunction and caspase-9 and -3 activation [45]. In addition, a recent report suggested that DMSO sensitized human myeloid leukemia cells to death-receptor-mediated apoptosis through mitochondrial membrane depolarization [46]. In our previous study, we demonstrated that HLJ1 could promote UV-induced apoptosis through enhancing JNK and caspase-3 activation in NSCLC [26]. Moreover, HLJ1 serves as a novel target of caspase-3 that is degraded at a late stage of apoptosis. Our data in the present study also showed that DMSO treatment induced the expression of HLJ1 and caused marked PARP cleavage after UV exposure, ultimately leading to increased cancer cell apoptosis. Taken together, these data suggest that the induction of sensitivity to UV irradiation by treatment with DMSO is mediated by HLJ1 upregulation in human lung cancer cells. However, whether HLJ1 plays a role in the mitochondrial pathway is still unknown. Further studies will be needed to clarify the relationship between HLJ1 and mitochondrial dysfunction in the apoptosis pathway. In addition, our results suggested that the concentration of DMSO used as the drug solvent would lead to expression of some genes (0.1% DMSO, Figure S1) and this issue should be noticed and considered in the medical therapeutic strategy. We also provide some clues that appropriate concentration of DMSO used under lethal dose may synergistically improve the effects of radiotherapy and chemotherapy in lung cancers.

In summary, our results suggest that DMSO is an important stimulator of the tumor suppressor protein HLJ1 through JunB and JunD activation in the highly invasive lung adenocarcinoma CL1–5 cells. This study is an important contribution both for the knowledge of the regulation, function and biomedical relevance of the tumor suppressor protein HLJ1 and for the understanding of the biological effects and mechanisms induced by DMSO. Our results also implied that DMSO may serve as a lead compound or ligand for the development of anticancer drugs that induce HLJ1 expression and regulate cell migration/invasion and cell proliferation-related pathways. These efforts will help us to develop not only the novel anti-cancer drugs for lung cancer progression but also new therapeutic strategies for the disease. For instance, a therapeutic strategy combining both induced expression of HLJ1 by DMSO-derived analogs and irradiation would synergistically increase the efficacy of radiotherapy and prolong lung cancer patient survival.

## Supporting Information

Figure S1 **DMSO induces HLJ1 expression in different lung adenocarcinoma cells in concentration-dependent manners.** Concentration-dependent DMSO-induced HLJ1 expression at the protein level was determined by Western blot analysis in A549 (A) and H1299 (B) cells. α-tubulin was an internal control for protein loading and transfer.
(TIF)

Figure S2 **Ethanol has no effect on HLJ1 induction in CL1–5 cells.** Western blot analysis reveals that HLJ1 protein expression was not induced by ethanol under various concentrations tested (0.1–5%, v/v) after 48 h incubation. β-actin was an internal control for protein loading and transfer.
(TIF)
