# Supplementary figures and images for: Dimethyl Sulfoxide Promotes the Multiple Functions of the Tumor Suppressor HLJ1 through Activator Protein-1 Activation in NSCLC Cells
Source: PLoS One. 2012 Apr 17;7(4):e33772. doi: 10.1371/journal.pone.0033772 (PMC3328470; doi:10.1371/journal.pone.0033772)

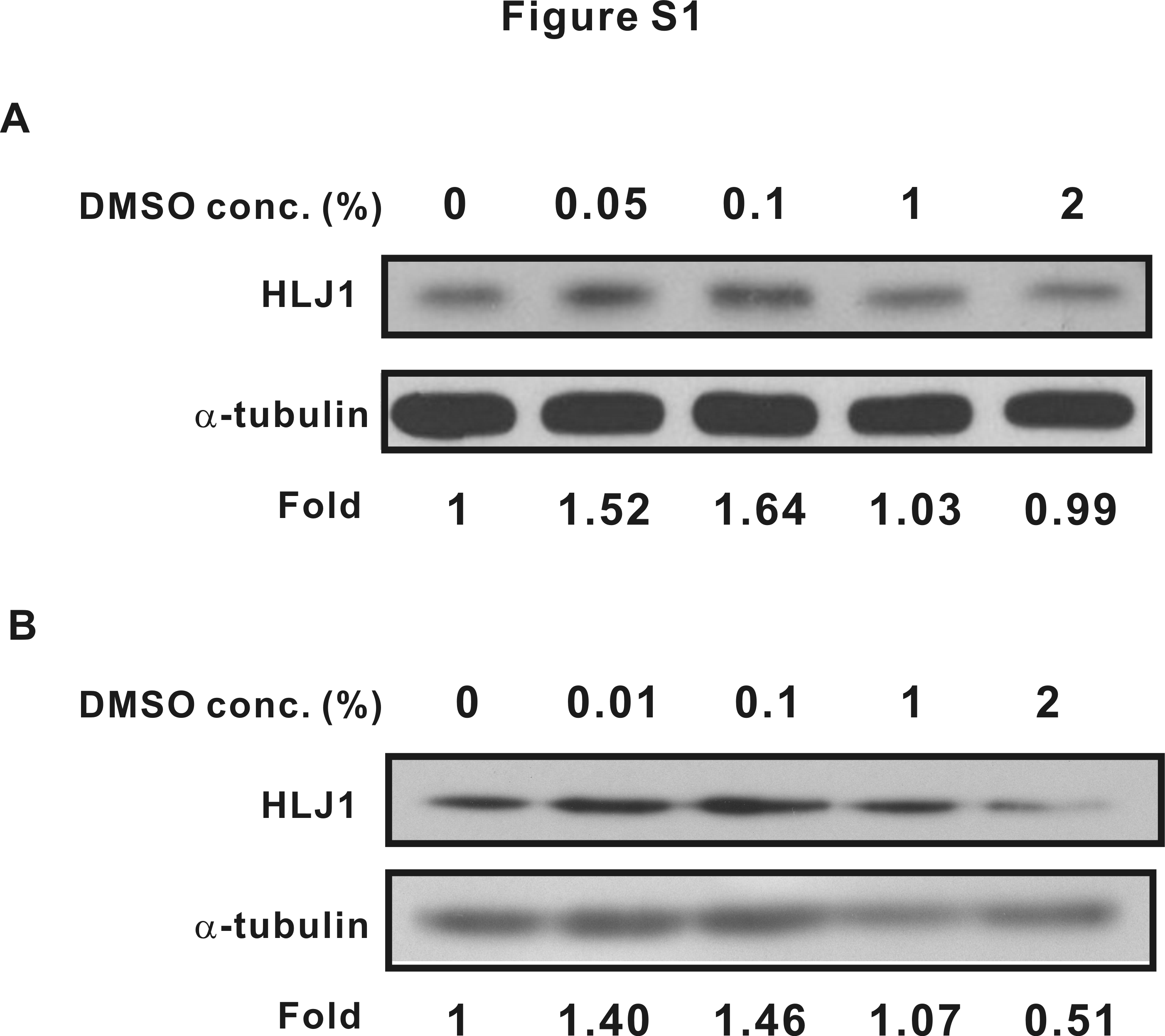

Supplement: Figure S1 — DMSO induces HLJ1 expression in different lung adenocarcinoma cells in concentration-dependent manners. Concentration-dependent DMSO-induced HLJ1 expression at the protein level was determined by Western blot analysis in A549 (A) and H1299 (B) cells. α-tubulin was an internal control for protein loading and transfer. (TIF) [file pone.0033772.s001.tif]

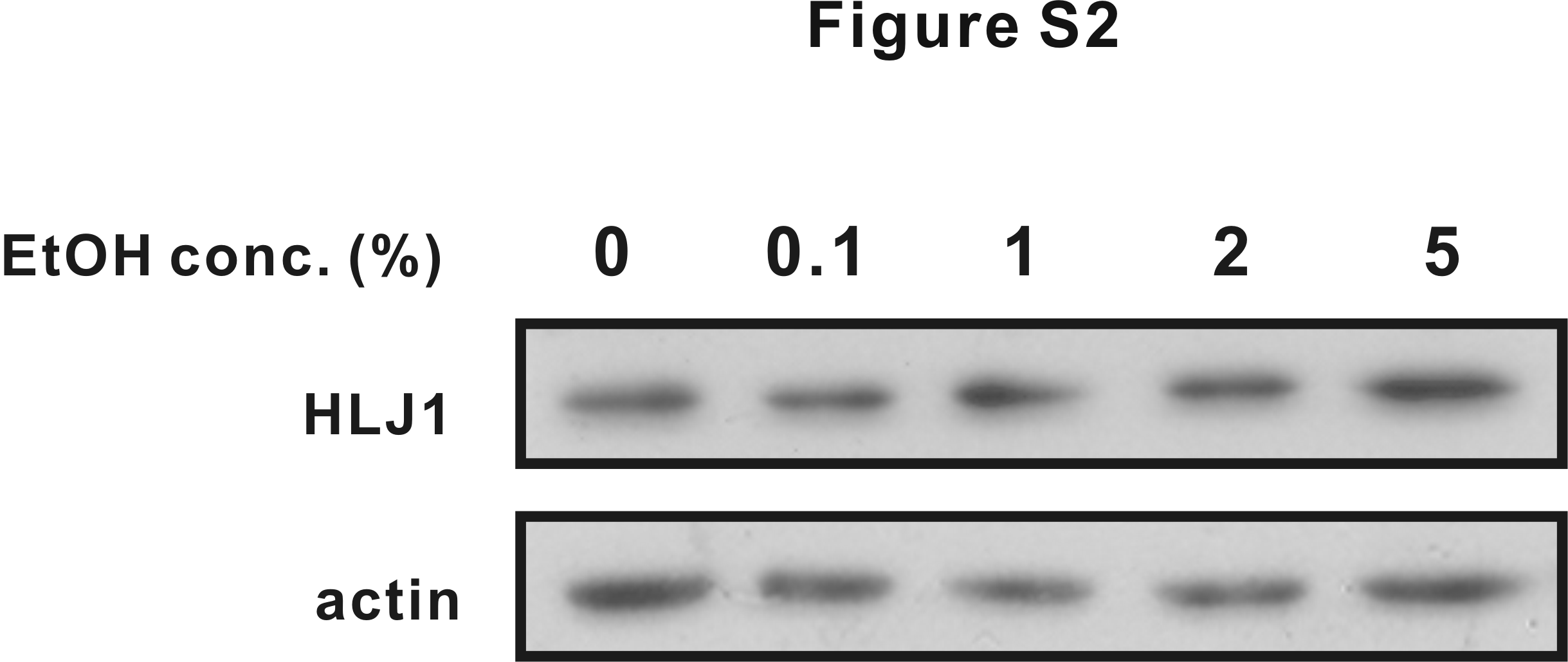

Supplement: Figure S2 — Ethanol has no effect on HLJ1 induction in CL1–5 cells. Western blot analysis reveals that HLJ1 protein expression was not induced by ethanol under various concentrations tested (0.1–5%, v/v) after 48 h incubation. β-actin was an internal control for protein loading and transfer. (TIF) [file pone.0033772.s002.tif]
